# Real-time reaction monitoring of silylation and acylation of carbohydrates with a conductometry set-up

**DOI:** 10.1016/j.carres.2026.109853

**Published:** 2026-02-16

**Authors:** Thitiphong Khamkhenshorngphanuch, Gustavo A. Kashiwagi, Yogesh Sutar, Sewan Theeramunkong, Nitipol Srimongkolpithak, Alexei V. Demchenko

**Affiliations:** a Department of Chemistry, Saint Louis University, 3501 Laclede Ave, St. Louis, MO, 63103, USA; b Department of General Education, Faculty of Sciences and Health Technology, Navamindradhiraj University, Bangkok, 10300, Thailand; c Thammasat University Research Unit in Drug, Health Product Development and Application (DHP-DA), Department of Pharmaceutical Sciences, Faculty of Pharmacy, Thammasat University, Pathum Thani, 12120, Thailand; d National Center for Genetic Engineering and Biotechnology (BIOTEC), National Science and Technology Development Agency (NSTDA), 113 Thailand Science Park, Pathum Thani, 12120, Thailand

## Abstract

The chemical synthesis of carbohydrates requires multiple protecting group manipulations, yet reaction monitoring is still largely relying on offline methods such as TLC, HPLC, or LC-MS. The absence of simple real-time reaction monitoring tools limits reproducibility, scalability, and integration into automated glycan synthesis platforms. Reported herein is the first application of electrical conductometry for monitoring of reactions involving protecting group manipulation with carbohydrates *in situ*. In particular, silylation and acylation reactions were used as representative cases to demonstrate how well conductivity profiles correlate with reaction progress. It was showcased that plateaus in conductivity profile coincides with complete substrate consumption, whereas variations in electrophiles and bases produced characteristic conductivity signatures. This work establishes conductometry as a low-cost, non-invasive, and accessible process analytical technology for carbohydrate chemistry. Beyond its immediate use in academic laboratories, the method holds promise for integration into automated and continuous-flow systems, ultimately advancing real-time monitoring, intervention, control, reproducibility in glycan synthesis.

## 1. Introduction

The chemical synthesis of carbohydrates and glycans remains a challenging task due to their structural complexity, which necessitates extensive protecting group manipulations, a deep understanding of orthogonality, and specialized expertise [1–6]. Over the past decades, automated technologies have been developed to simplify these processes, with several platforms approaching a mature state of the art [7–19]. In pursuit of more accessible solutions, our laboratory has introduced HPLC-based automated platform (HPLC-A) capable of automating building block synthesis, glycosylation, glycan assembly, and purification [20–26]. This platform allow researchers perform carbohydrate synthesis and purification with basic chromatographic equipment and minimal experimental training, thereby lowering the technical barrier to enter the highly specialized field of carbohydrate chemistry.

Despite such technological advances, a critical limit persists: the lack of reliable and straightforward methods to monitor reactions in real time. Current analytical approaches including NMR, IR, UV-vis, and mass spectrometry have been applied to reaction monitoring [27–30]. Nevertheless, all these approaches are costly, technically demanding, and/or impractical for continuous real-time analysis in automated technologies or educational settings [31–34]. This shortcoming frequently results in reduced reproducibility, difficulties of scaling the reactions, and inconsistencies in yields or regioselectivity when reaction progress cannot be tracked directly. Therefore, there is a strong need for an accessible, inexpensive, and non-invasive monitoring technique specifically tailored to synthetic carbohydrate chemistry.

**Scheme 1.**
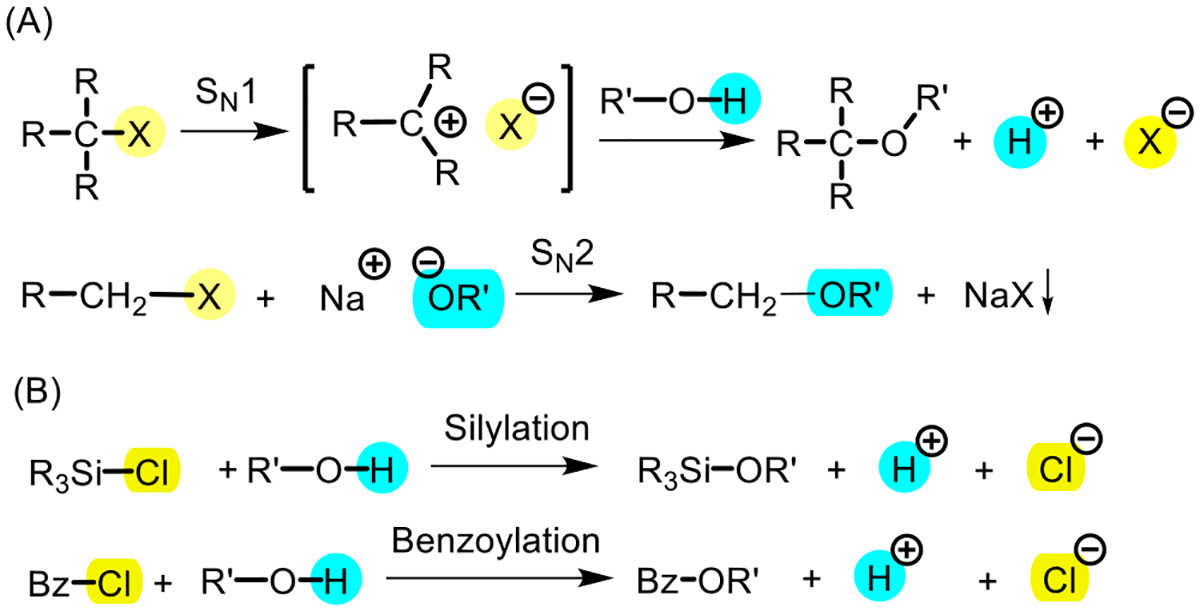
Conductivity shifts in organic reactions.

Conductometry represents a simple and widely available analytical tool, primarily used to follow chemical or enzymatic reactions in aqueous systems [35]. Conductometric monitoring has been successfully applied in the solid-phase peptide synthesis for amino acid coupling and protecting group removal reactions [36–39]. Another useful aspect is that conductivity changes can reveal mechanistic details of organic reactions, such as S_N_1 and S_N_2 processes in nonaqueous solutions, wherein conductivity increases with ion generation (S_N_1) or decreases due to precipitation of salts (NaX, S_N_2, Scheme 1A) [40]. These observations suggest that conductometry can serve as a sensitive probe for reaction dynamics. Nevertheless, its application in organic synthesis has been limited due to the low conductivity of non-ionic organic compounds [41]. To date, no reports describing the application of conductometry to monitor reactions with carbohydrates have yet emerged.

Herein, we present the first study to establish and validate the use of conductometry as a real-time reaction monitoring method for silylation and acylation of carbohydrates (Scheme 1B). By demonstrating robust conditions under which conductivity profiles accurately reflect reaction progress, this work highlights conductometry as a complementary, low-cost process analytical technology. Beyond its immediate utility in academic laboratories or educational settings, this approach has the potential to enhance reproducibility, streamline optimization, and facilitate integration into automated glycan synthesizers and continuous-flow systems. Ultimately, the implementation of conductometry may contribute to advancing carbohydrate chemistry toward more reproducible, scalable, and industrially relevant practices.

## 2. Results and discussion

**Fig. 1.**
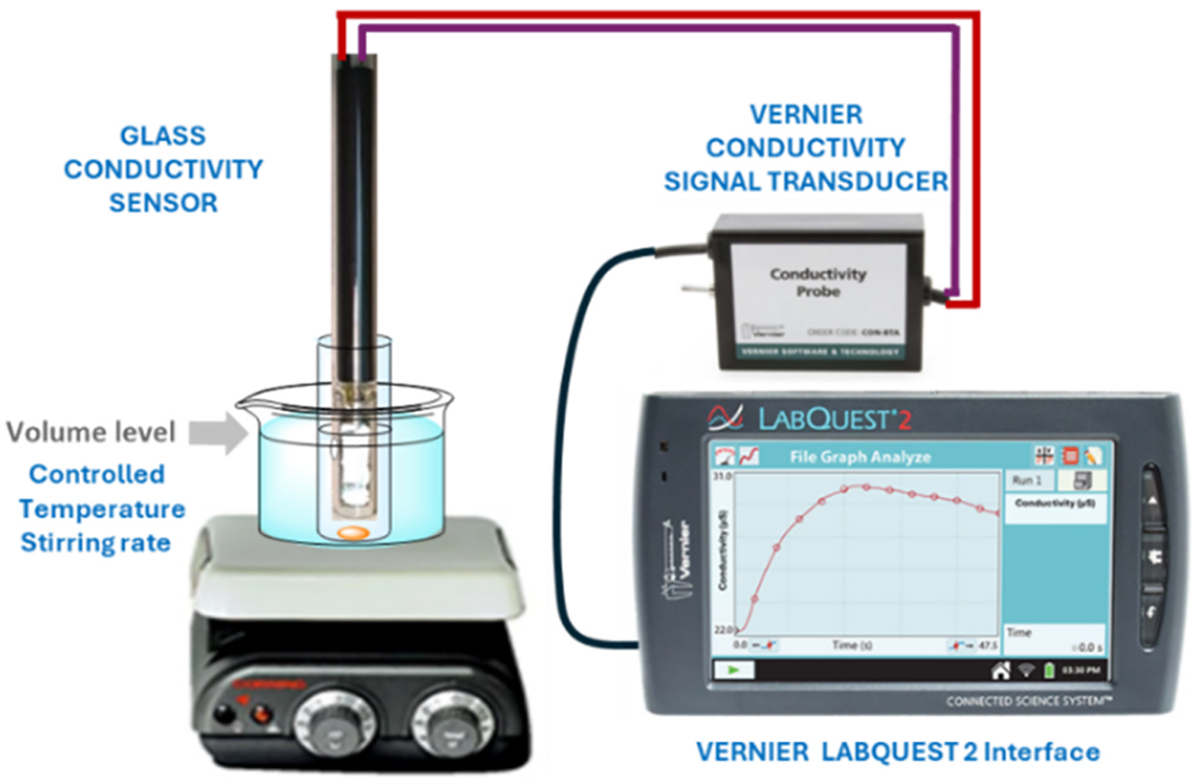
Experimental setup for real-time conductometric monitoring of reactions.

The conductometric setup depicted in Fig. 1 consisted of a Vernier LabQuest 2 Data Logger (LQ2-LE) equipped with a Vernier conductivity driver. For conductivity measurements, glass conductivity cells were employed: Van London 402 7G0C210 SKU: E8072 (cell constant 0.1 cm^−1^) and Sentek Ltd K40 SKU: 240–75 (cell constant 0.1 cm^−1^). Both models are dip-type cells, constructed with a glass body and platinum electrodes. Prior to each use, the cell was rinsed with acetone and dried with a stream of hot air. Calibration of the electrodes was performed using standard KBr solutions supplied by Vernier Co. We selected the Vernier system due to its widespread use in academic and teaching laboratories. These instruments offer versatility, operational safety, and straightforward data acquisition practices. Since the commercial Vernier conductivity probe is not compatible with organic solvents, it was replaced with a glass cell fitted with inert electrodes. The reaction vessel was maintained at a constant temperature using a water bath. All reactions were performed in a standard laboratory test tube, cut 1.5 inches from the bottom to facilitate the introduction of reagents. Given the small reaction scale, no measurable heat changes were observed during the assays. We note that small variation of the temperature (plus/minus 1–5 °C) could be easily tolerated because it did not impact the experimental set up. In all the experiments, conductivity was recorded every 10 s for a period of 1 to 4 h, depending on the experiment, upon the addition of the electrophile or the nucleophile. The conductivity probe was always maintained in a fixed position inside the test tube containing the reaction mixture, while the test tube was kept at constant temperature using a thermostatic bath (Fig. 1).

The stirring velocity was maintained at 700 RPM, during the data recording. However, preliminary experiments demonstrated no appreciable changes or disruption of the signal stability in the range of 500 to 800 RPM. Reactions were monitored by conductometric measurements in parallel with thin-layer chromatography (TLC).

To evaluate the feasibility of conductometric monitoring in common reactions used in carbohydrate chemistry, we first optimized general experimental parameters such as concentration, temperature, and stirring rate to ensure that conductivity values remained within the instrument’s detection range and produced reproducible signals. The silylation reaction involves reaction of the hydroxyl group as the nucleophile with a silyl chloride electrophile in the presence of a base. In addition to the formation of the silyl ether product, the reaction generates chloride anions and protonated bases, which contribute to measurable changes in conductivity. Thus, the conductivity profile was expected to increase as ionic species accumulate in the reaction mixture. This should eventually reach the plateau, no changes in conductivity, when the reaction nears completion.

**Fig. 2.**
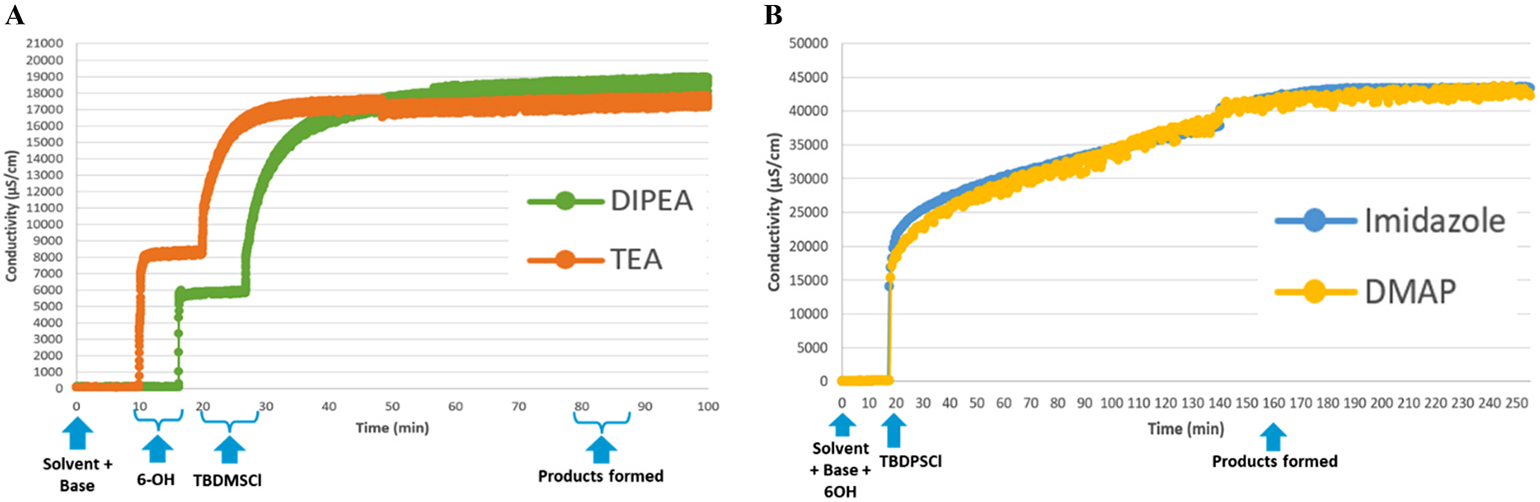
Conductivity monitoring of silylations at 700 RPM at rt.

Systematic evaluation revealed that the choice of base, solvent, and particularly the order of reagent addition exerted a strong influence on both conductivity profiles and overall reaction outcomes. The key results of optimization of silylation reactions are summarized in Table 1. The benchmark experiment for the reaction of 6-OH derivative **1** with TBDMSCl was inspired by our recent automated synthesis protocol wherein 6-*O*-TBDMS derivative **2** was obtained in 81% yield (entry 1) [25]. We first performed the monitored experiments whereat carbohydrate nucleophile **1** was introduced after TBDMSCl and base. Regardless of the base, catalytic additive, or solvent, these reactions proceeded very slowly, and were still incomplete even after 24 h (40–80% conversion, entries 2–6). In contrast, when TBDMSCl was added last to the pre-mixed solution of carbohydrate and the base, the conductivity increased more rapidly (entries 7 and 8 and Fig. 2A). Both traces reached the plateau within 60 min after the addition of the base. Thus, in the presence of DIPEA (entry 7), TBDMSCl was added at 25 min time point, and reaction was completed by 85 min, a timepoint whereat no changes in conductivity were no longer detected. Similarly, in the presence of TEA (entry 8), TBDMSCl was added at 20 min time point, and reaction was completed by 80 min. TLC analysis at these plateaus confirmed complete consumption of the starting material and the appearance of a single, less polar product consistent with the expected silyl ether **2**. All products in successful reactions have been isolated and fully characterized.

We next investigated the effect of a bulkier electrophile, *tert*-butyldiphenylsilyl chloride (TBDPSCl). As anticipated, reactions with TBDPSCl were slower, and we initially investigated the reagent ratios to determine the most favorable reaction conditions. Upon varying the amounts of TBDPSCl (1.5–5.0 equiv) and base (1.0–3.0 equiv), we determined that higher reagent loading produced larger conductivity responses and shorter reaction times for the synthesis of 6-*O*-TBDPS derivative **3** (see the SI for additional details) Complete conversion was observed with 5.0 equiv of the electrophile and 3.0 equiv of the base (Imidazole, entry 9 and DMAP, entry 10) within ~140 min (Fig. 2B). The conductivity traces again exhibited a clear plateau that coincided with reaction completion, as confirmed by TLC, demonstrating that the method is applicable to electrophiles of different steric demands.

Overall, these results demonstrate that conductometric monitoring provides a sensitive and non-invasive means of following silylation reactions with carbohydrates in real time. The conductivity plateau correlates directly with reaction completion and yield, while differences in base, solvent, electrophile, and reagent stoichiometry can be reflected in the conductivity profiles. These findings establish conductometry as a valuable complementary technique to TLC, capable of providing continuous feedback on the progress of silylation reactions under diverse conditions.

**Fig. 3.**
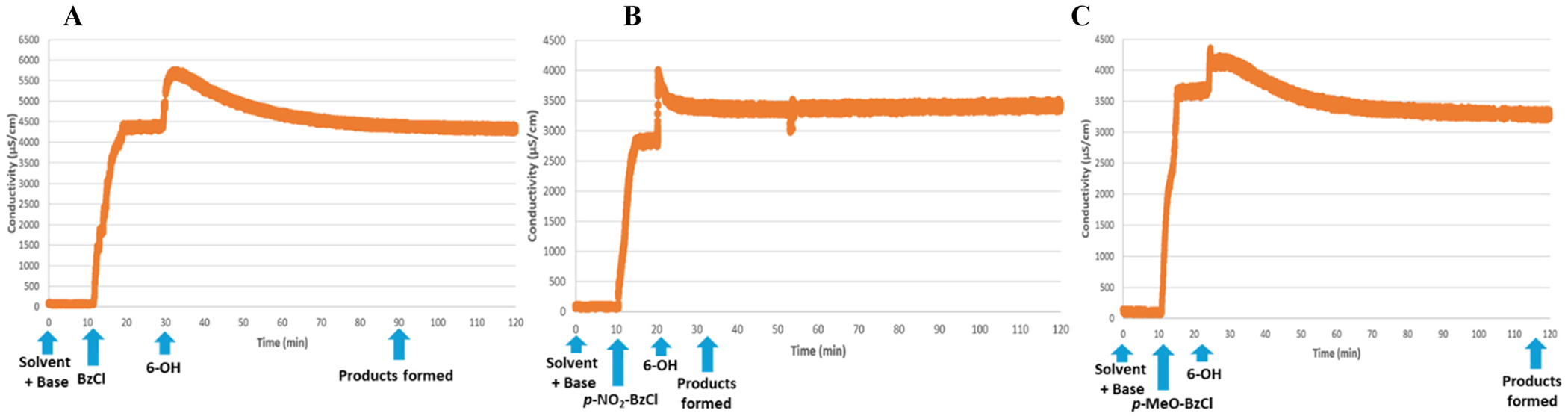
Conductivity monitoring of acylation at 700 RPM at 45 °C.

Systematic evaluation of benzoylation reaction revealed that the choice of base, solvent, and particularly the reaction temperature exerted a strong influence on both conductivity profiles and overall reaction outcomes. The key results of optimization of benzoylation reactions are summarized in Table 2. The benchmark experiment for the reaction of 6-OH derivative **1** with BzCl was inspired by our recent automated synthesis protocol wherein 6-*O*-benzoylated derivative **4** was obtained in 81% yield (entry 1) [25]. We first performed the monitored experiments whereat reactions were performed at rt. Regardless of the base, catalytic additive, or solvent, these reactions proceeded slowly (2–5 h, and were still incomplete (40–80% conversion, entries 3–6). We then conducted reactions at 45 °C to enhance reaction kinetics. Under these conditions, benzoylation smoothly proceeded to completion within 1 h in the absence of DMF (entry 8 and Fig. 3A); in contrast, reaction performed in the presence of DMF did not reach full conversion probably due to BzCl-DMF interactions (entry 7) [42]. The conductometric profile of benzoylation reactions displayed an initial rapid increase in conductivity within the first 2–4 min after the addition of benzoyl chloride, which we attribute to the formation of ionic intermediates such as pyridinium chloride and other protonated bases generated upon reaction of benzoyl chloride with pyridine or DMAP. Following this spike, the signal gradually decreased and approached a plateau, indicative of the system reaching an ionic equilibrium between the consumed electrophile, the generated ionic byproducts, and the final benzoylated product.

We then performed acylation with para-substituted benzoyl chlorides. With *p*-nitrobenzoyl chloride (entry 9 and Fig. 3B), the conductivity curve reached a steady state within 10 min, which was consistent with accelerated electrophilic reactivity. TLC at that point confirmed full conversion and a single UV-active spot corresponding to *p*-nitrobenzoylated product **5**. In contrast, the electron-donating *p*-methoxybenzoyl chloride required over 100 min to reach a plateau, reflecting its lower electrophilicity. The corresponding TLC analysis showed near-quantitative formation of *p*-methoxybenzoylated product **6**, consistent with the conductivity profile (entry 10, Fig. 3C). These observations validate that conductometric monitoring can distinguish between reaction kinetics driven by electronic effects of the acylating reagent.

Taken together, these results demonstrate that conductometry is a robust and non-invasive method for monitoring benzoylation reactions of carbohydrate derivatives. It not only provides rapid feedback on the reaction endpoint but also sensitively reports on electrophile reactivity trends. This highlights its potential as a process analytical technology tool that can be integrated into both batch and flow carbohydrate chemistry for efficient reaction optimization and control.

## 3. Conclusions

In this study, we demonstrated that conductometry can serve as a simple, non-destructive, and real-time tool to monitor key protecting group reactions in carbohydrate chemistry. Both silylation and acylation reactions displayed characteristic conductivity profiles, where plateau regions correlated with complete substrate consumption and final yields confirmed by TLC. These findings highlight the utility of conductometry not only as a complementary method to TLC but also as a rapid screening technique to optimize reaction conditions. While conductivity measurements in organic solvents are sensitive to parameters such as solvent polarity, temperature, and ionic byproducts, our results show that reproducible profiles can be obtained under carefully controlled conditions. Importantly, this is the first demonstration of conductometry applied to protecting group manipulations in carbohydrate chemistry, establishing proof-of-concept for its broader application. Looking forward, extending this approach to glycosylation and continuous-flow synthesis could enhance reproducibility, facilitate automation, and align carbohydrate chemistry with modern process analytical technology frameworks.

## 4. Experimental

### General methods.

The compounds were detected by examination under UV light and by charring with 10% sulfuric acid in methanol. Column chromatography was performed on silica gel 60 (70–230 mesh), and reactions were monitored by TLC on Kieselgel 60 F254. Solvents were removed under reduced pressure at <40 °C. *tert*-Butyldimethylsilyl chloride (TBDMSCl), *tert*-butyldiphenylsilyl chloride (TBDPSCl), benzoyl chloride (BzCl), *p*-methoxybenzoyl chloride, and *p*-nitrobenzoyl chloride were used as electrophilic reagents. Imidazole and triethylamine were employed as bases. Dichloromethane (DCM), chloroform (CHCl_3_), acetonitrile (MeCN), and dimethylformamide (DMF) were obtained from commercial suppliers and (all ACS grade) used without further purification. Optical rotations were measured using a Jasco polarimeter. ^1^H NMR spectra were recorded in CDCl_3_ at 400 or 700 MHz. ^13^C NMR spectra were recorded in CDCl_3_ at 101 or 176 MHz. The ^1^H NMR chemical shifts are given relative using internal tetramethyl silane (TMS, *δ*_H_ = 0 ppm) standard for solutions in CDCl_3_ and. The ^13^C NMR chemical shifts are referenced to the central signal of CDCl_3_ (*δ*_C_ = 77.00 ppm) for solutions in CDCl_3_. Accurate mass spectrometry determinations (HRMS) were performed using Agilent 6230 ESI TOF LCMS mass spectrometer.

### General procedures for silylation.

Two different reagent addition orders were investigated. *Addition method A*. Silyl chloride was added to a test tube containing a solvent or a mixture of solvents (3.0 mL) and a base with or without catalytic additive. Once the conductivity trace has reached a constant value, carbohydrate nucleophile (**1**, 0.13 mmol,1.0 equiv) was added as a solid, and the reaction was monitored for the time listed in Table 1. The addition of **1** as the solid caused minimal changes to the volume of the reaction mixture. *Addition method B*. Carbohydrate nucleophile (**1**, 1.0 equiv) was added to a test tube containing a solvent or a mixture of solvents (3.0 mL) and a base with or without catalytic additive. Once the conductivity trace has reached a constant value, silyl chloride was added, and the reaction was monitored for the time listed in Table 1. TLC analyses were performed at key points along the electrical conductivity curves to elucidate the electrical behavior and monitor the reaction progress. After that, the reaction mixture was diluted with DCM (10 mL) and washed with sat. aq. NaHCO_3_ and water (2 × 10 mL). The organic phase was separated, dried with sodium sulfate, and concentrated under reduced pressure. The residue was purified by column chromatography on silica gel (ethyl acetate-hexane gradient elution) to afford the corresponding products (**2** or **3**).

### General procedure for benzoylation.

Aryl chloride was added to a test tube containing a solvent or a mixture of solvents (3.0 mL) and a base with or without catalytic additive. Once the conductivity trace has reached a constant value, carbohydrate nucleophile (**1**, 0.13 mmol, 1.0 equiv) was added as a solid, and the reaction was monitored for the time listed in Table 2. The addition of **1** as the solid caused minimal changes to the volume of the reaction mixture. TLC analyses were performed at key points along the electrical conductivity curves to elucidate the electrical behavior and monitor the reaction progress. After that, the reaction mixture was quenched with MeOH, and solvent was evaporated using rotary evaporator. The reaction mass was extracted with DCM (10 mL), and washed with sat. aq. NaHCO_3_, and water (2 × 10 mL). The organic phase was separated, dried with sodium sulfate, and concentrated under reduced pressure. The residue was purified by column chromatography on silica gel (ethyl acetate-hexane gradient elution) to afford the corresponding products (**4**–**6**).

**Methyl 2,3,4-tri-*O*-benzyl-α-d-glucopyranoside (1)** was synthesized as reported previously and its analytical data was in accordance with that previously described [43].

**Methyl 2,3,4-tri-*O*-benzyl-6-*O*-*tert*-butyldimethylsilyl-α-d-glucopyranoside (2)**. Analytical data was in accordance with that previously described [25].

**Methyl 2,3,4-tri-*O*-benzyl-6-*O*-*tert*-butyldiphenylsilyl-α-d-glucopyranoside (3)**. The title compound was obtained from **1** (60 mg, 0.13 mmol) in 2.2 h (85 mg, 94 %) as white crystals. Analytical data for **3**: m. p. +109.3–109.8 °C; R_*f*_ 0.40 (ethyl acetate/hexane, 0.15/0.85, v/v); ${\left[\alpha \right]}_{\text{D}}^{24.9}+12.4$ (*c* = 1.0, CHCl_3_); ^1^H NMR (400 MHz, CDCl_3_): *δ* 7.73–7.64 (m, 4H, aromatic), 7.45–7.20 (m, 19H, aromatic), 7.18–7.10 (m, 2H, aromatic), 4.98 (d, *J* = 10.8 Hz, 1H, C*H*Ph), 4.87 (d, *J* = 10.8 Hz, 1H, C*H*Ph), 4.83 (d, *J* = 10.8 Hz, 1H, C*H*Ph), 4.81 (d, *J* = 12.1 Hz, 1H, C*H*Ph), 4.70 (d, *J* = 12.1 Hz, 1H, C*H*Ph), 4.66 (d, *J* = 3.6 Hz, 1H, H-1), 4.60 (d, *J* = 10.8 Hz, 1H, C*H*Ph), 4.01 (dd, *J* = 9.6, 9.0 Hz, 1H, H-3), 3.91–3.82 (m, 2H, H-6a, 6b), 3.69 (ddd, *J* = 10.0, 3.1 Hz, 1H, H-5), 3.61 (dd, *J* = 10.0, 9.0 Hz, 1H, H-4), 3.55 (dd, *J* = 9.6, 3.6, 3.6 Hz, 1H, H-2), 3.37 (s, 3H, OCH_3_), 1.04 (s, 9H, ^*t*^Bu) ppm; ^13^C NMR (CDCl_3_, 101.0 MHz): *δ* 138.9, 138.4 (x 2), 135.9 (x 2), 135.8 (x 2), 133.7, 133.4, 129.7 (x 2), 128.6 (x 4), 128.5 (x 2), 128.3 (x 2), 128.2 (x 2), 128.0 (x 3), 127.8 (x 4), 127.7 (x 2), 98.0 (C-1), 82.4 (C-3), 80.4 (C-2), 78.0 (C-4), 76.0 (*C*H_2_Ph), 75.2 (*C*H_2_Ph), 73.5 (*C*H_2_Ph), 71.6 (C-5), 63.1 (C-6), 55.0 (OCH_3_), 26.9 (C(*C*H_3_)_3_), 19.4 (*C*(CH_3_)_3_); HR- LCMS: *m/z* calcd for C_35_H_35_NO_9_Na^+^ [M+Na]^+^ 725.3274, found 725.3273.

**Methyl 6-*O*-benzoyl-2,3,4-tri-*O*-benzyl-α-d-glucopyranoside (4)**. Analytical data was in accordance with that previously described [25].

**Methyl 2,3,4-tri-*O*-benzyl-6-*O*-*p*-nitrobenzoyl-α-d-glucopyranoside (5).** The title compound was obtained from **1** (60 mg, 0.13 mmol) in 10 min (72.1 mg, 91%) as white crystals. Analytical data for **5**: m. p. +82.4–83.3 °C; R_*f*_ 0.50 (ethyl acetate/hexane, 1/3, v/v); ${\left[\alpha \right]}_{\text{D}}^{24.9}+32.9$ (*c* = 1.0, CHCl_3_); ^1^H NMR (700 MHz, CDCl_3_): *δ* 8.29–8.24 (m, 2H, aromatic), 7.40–7.25 (m, 17H, aromatic), 5.02 (d, 1H, *J* = 10.8 Hz, C*H*Ph), 4.92 (d, 1H, *J* = 11.1 Hz, C*H*Ph), 4.84 (d, 1H, *J* = 10.8 Hz, C*H*Ph), 4.81 (d, 1H, *J* = 12.1 Hz, C*H*Ph), 4.67 (d, 1H, *J* = 12.1 Hz, C*H*Ph), 4.67 (d, 1H, *J* = 11.1 Hz, C*H*Ph), 4.61 (d, 1H, *J* = 3.6 Hz, H-1), 4.44 (dd, 1H, *J* = 11.5, 2.3 Hz, H-6a), 4.37 (dd, 1H, *J* = 11.5, 4.7 Hz, H-6b), 4.03 (dd, 1H, *J* = 9.6, 8.8 Hz, H-3), 3.88 (ddd, 1H, *J* = 10.1, 4.7, 2.3 Hz, H-5), 3.56 (dd, 1H, *J* = 9.6, 3.6 Hz, H-2), 3.52 (dd, 1H, *J* = 10.1, 8.8 Hz, H-4), 3.40 (s, 3H, OCH_3_) ppm; ^13^C NMR (CDCl_3_, 176 MHz): *δ* 155.6, 152.4, 145.5, 138.7, 138.1, 137.9, 128.7 (x 4), 128.6 (x 2), 128.3 (x 2), 128.2 (x 3), 128.1 (x 3), 127.9, 125.4 (x 2), 121.8 (x 2), 98.3 (C-1), 82.1 (C-3), 79.9 (C-2), 77.1 (C-4), 76.0 (*C*H_2_Ph), 75.2 (*C*H_2_Ph), 73.6 (*C*H_2_Ph), 68.5 (C-5), 67.9 (C-6), 55.6 (OCH_3_); HR-LCMS: *m/z* calcd for C_35_H_35_NO_9_K^+^ [M+K]^+^ 652.1949, found 652.1932.

**Methyl 2,3,4-tri-*O*-benzyl-6-*O*-*p*-methoxybenzoyl-α-d-glucopyranoside (6).** The title compound was obtained from **1** (60 mg, 0.13 mmol) in 1.7 h (72.2 mg, 93%) as a clear syrup. Analytical data: R_*f*_ 0.55 (ethyl acetate/hexane, 1/3, v/v); ${\left[\alpha \right]}_{\text{D}}^{24.8}+48.3$ (*c* = 1.0, CHCl_3_); ^1^H NMR (700 MHz, CDCl_3_): *δ* 7.96–7.95 (m, 2H, aromatic), 7.38–7.24 (m, 15H, aromatic), 6.90–6.89 (m, 2H, aromatic), 5.01 (d, *J* = 10.7 Hz, 1H, C*H*Ph), 4.90 (d, *J* = 10.8 Hz, 1H, C*H*Ph), 4.85 (d, *J* = 10.7 Hz, 1H, C*H*Ph), 4.81 (d, *J* = 12.1 Hz, 1H, C*H*Ph), 4.68 (d, *J* = 12.1 Hz, 1H, C*H*Ph), 4.62 (d, *J* = 3.6 Hz, 1H, H-1), 4.61 (d, *J* = 10.9 Hz, 1H, C*H*Ph), 4.52 (dd, *J* = 12.0, 2.2 Hz, 1H, H-6a), 4.45 (dd, *J* = 12.0, 4.8 Hz, 1H, H-6b), 4.05 (dd, *J* = 9.6, 9.0 Hz, 1H, H-3), 3.94 (ddd, *J* = 10.0, 4.8, 2.2 Hz, 1H, H-5), 3.85 (s, 3H, OCH_3_), 3.59 (dd, *J* = 10.0, 9.0 Hz, 1H, H-4), 3.57 (dd, *J* = 9.6, 3.6 Hz, 1H, H-2), 3.38 (s, 3H, OCH_3_) ppm; ^13^C NMR (176 MHz, CDCl_3_): *δ* 166.1, 163.6, 138.7, 138.2, 137.9, 131.8 (x 2), 128.6 (x 6), 128.2 (x 6), 128.1, 128.0, 127.9, 122.4, 113.7 (x 2), 98.1 (C-1), 82.2 (C-3), 80.2 (C-2), 77.8 (C-4), 76.1 (*C*H_2_Ph), 75.3 (*C*H_2_Ph), 73.5 (*C*H_2_Ph), 68.9 (C-5), 63.3 (C-6), 55.5 (OCH_3_), 55.3 (OCH_3_); HR- LCMS: *m/z* calcd for C_36_H_38_O_8_Na^+^ [M+Na]^+^ 621.2464, found 621.2459.

## Supplementary Material

SI

## Figures and Tables

**Table 1:** Effect of electrophile, solvents, catalysts, and bases on the monitored silylation reaction.

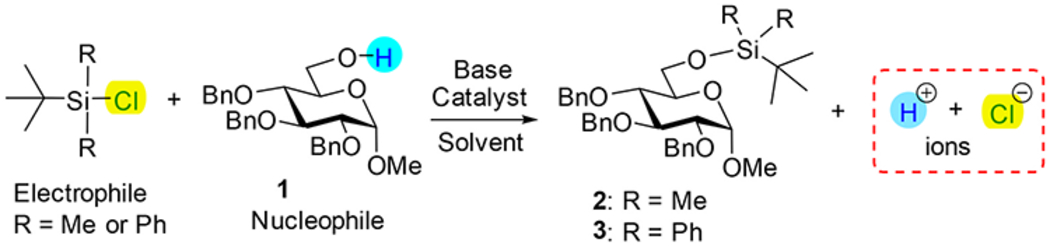						
Entry	Base (equiv)	Catalyst (equiv)	Electrophile (equiv)	Solvent(s)	Temperature, time	Product, yield^(a)^
1^(b)^	TEA (4.5)	Imidazole (1.1)	TBDMSCl (4.0)	DCM:MeCN (3:2)	rt, 5.0 h	**2**, 81%^25^
2^(b)^	Imidazole (1.5)	None	TBDMSCl (1.5)	MeCN:CHCl_3_ (3:2)	rt, >24 h	**2**, 40%
3^(b)^	Imidazole (1.5)	None	TBDMSCl (1.5)	MeCN	rt, >24 h	**2**, 80%
_4_^(b)^	TEA (1.5)	DMAP (0.1)	TBDMSCl (1.5)	CHCl_3_:DMF (3:2)	rt, >24 h	**2**, 40%
5^(b)^	DMAP (1.0)	None	TBDMSCl (1.5)	CHCl_3_:DMF (3:2)	rt, >24 h	**2**, 60%
6^(b)^	TEA (1.5)	DMAP (0.1)	TBDMSCl (1.5)	MeCN:DMF (3:2)	rt, >24 h	**2**, 60%
7^(c)^	DIPEA (1.5)	None	TBDMSCl (1.5)	MeCN:DMF (3:2)	rt, 1.0 h	**2**, quant
8^(c)^	TEA (1.5)	None	TBDMSCl (1.5)	MeCN:DMF (10:1)	rt, 1.0 h	**2**, quant
9^(c)^	Imidazole (3.0)	None	TBDPSCl (5.0)	MeCN:DMF (3:2)	rt, 2.2 h	**3**, quant
10^(c)^	DMAP (3.0)	None	TBDPSCl (5.0)	MeCN:DMF (3:2)	rt, 2.2 h	**3**, quant

All reactions were performed using 0.13 mmol scale and 3.0 mL of the reaction solvent (or solvent mixture).

a estimated based on TLC.

b Addition Method A was followed.

c Addition Method B was followed (see Fig. 2 and the experimental sect^a^on for details).

**Table 2:** Effect of electrophile, solvents, catalysts, and bases on the monitored benzoylation reaction.

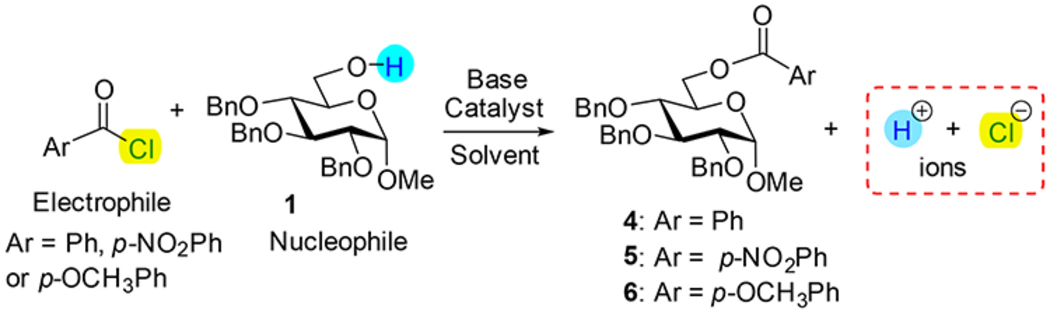						
Entry	Base (equiv)	Catalyst (equiv)	Electrophile (equiv)	Solvent(s)	Temp. (time)	Product, yield^(a)^
1	TEA (1.5)	DMAP (0.2)	BzCl (1.6)	DCM	rt, 21 h	**4**, 81% [25]
2	TEA (1.0)	None	BzCl (1.0)	DCM	rt, 24 h	None
3	Pyridine (1.0)	DMAP (0.1)	BzCl (1.0)	DCM	rt, 4 h	**4**, 40%
4	Pyridine (1.0)	DMAP (0.1)	BzCl (1.0)	DCM/DMF (3:2)	rt, 2 h	**4**, 80%
5	Pyridine (1.5)	Imidazole (0.1)	BzCl (1.5)	DCM/DMF (3:2)	rt, 5 h	**4**, 50%
6	TEA (1.5)	DMAP (0.1)	BzCl (1.5)	DCM/DMF (1:1)	rt, 5 h	**4**, 50%
7	None	DMAP (0.1)	BzCl (1.5)	Pyridine/CHCl_3_/DMF (2:1:1)	45 °C, 5 h	**4**, 40%
8	None	DMAP (0.1)	BzCl (1.5)	Pyridine/CHCl_3_ (2:1)	45 °C, 1 h	**4**, quant
9	None	DMAP (0.1)	*p*-NO_2_BzCl (1.5)	CHCl_3_/pyridine (2:1)	45 °C, 10 min	**5**, quant
10	None	DMAP (0.1)	*p*-OMeBzCl (1.5)	CHCl_3_/pyridine (2:1)	45 °C, >100 min	**6**, quant

All reactions were performed using 0.13 mmol scale and 3.0 mL of solvent.

a estimated based on TLC.

## Data Availability

Data will be made available on request.

## Appendix A. Supplementary data

Supplementary data to this article can be found online at https://doi.org/10.1016/j.carres.2026.109853.
