# Supplementary material for: Real-time reaction monitoring of silylation and acylation of carbohydrates with a conductometry set-up
Source: Carbohydr Res. Author manuscript; Available in PMC 2026 May 1. (PMC13067880; doi:10.1016/j.carres.2026.109853)

Supporting Information

Real-time reaction monitoring of silylation and acylation of carbohydrates with a conductometry set-up

Gustavo A. Kashiwagi,[1,*] Thitiphong Khamkhenshorngphanuch,[1,2] Yogesh Sutar,[1] Sewan Theeramunkong,[3] Nitipol Srimongkolpithak,[4] and Alexei V. Demchenko[1,*]

[1] Department of Chemistry, Saint Louis University, 3501 Laclede Ave, St. Louis, MO 63103, USA

[2] Department of General Education, Faculty of Sciences and Health Technology, Navamindradhiraj University, Bangkok 10300, Thailand

[3] Thammasat University Research Unit in Drug, Health Product Development and Application (DHP-DA), Department of Pharmaceutical Sciences, Faculty of Pharmacy, Thammasat University, Pathum Thani, 12120, Thailand.

[4] National Center for Genetic Engineering and Biotechnology (BIOTEC), National Science and Technology Development Agency (NSTDA), 113 Thailand Science Park, Pathum Thani, 12120, Thailand.

** Correspondence: gustavokashiwagi@gmail.com; alexei.demchenko@slu.edu*

Contents:

Additional Experimental Results S2

NMR Spectra for all New Compounds S3

Additional Experimental Results

Figure S1. Conductivity of silylation reaction with TBSPSCl at different amounts of reagents

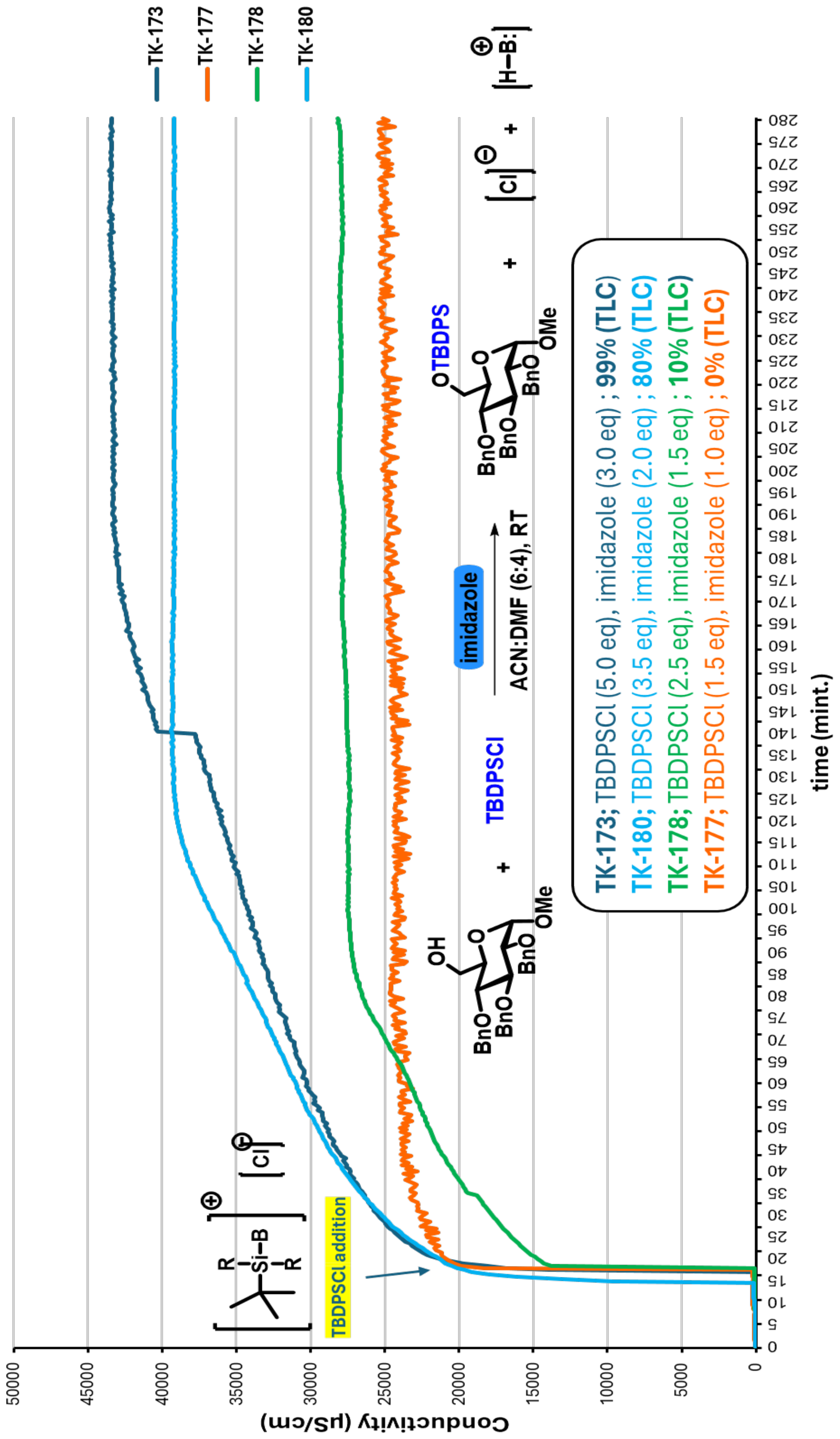

NMR Spectra for all New Compounds

Methyl 2,3,4-tri-*O*-benzyl-6-*O*-*tert*-butyldiphenylsilyl-α-D-glucopyranoside (3).

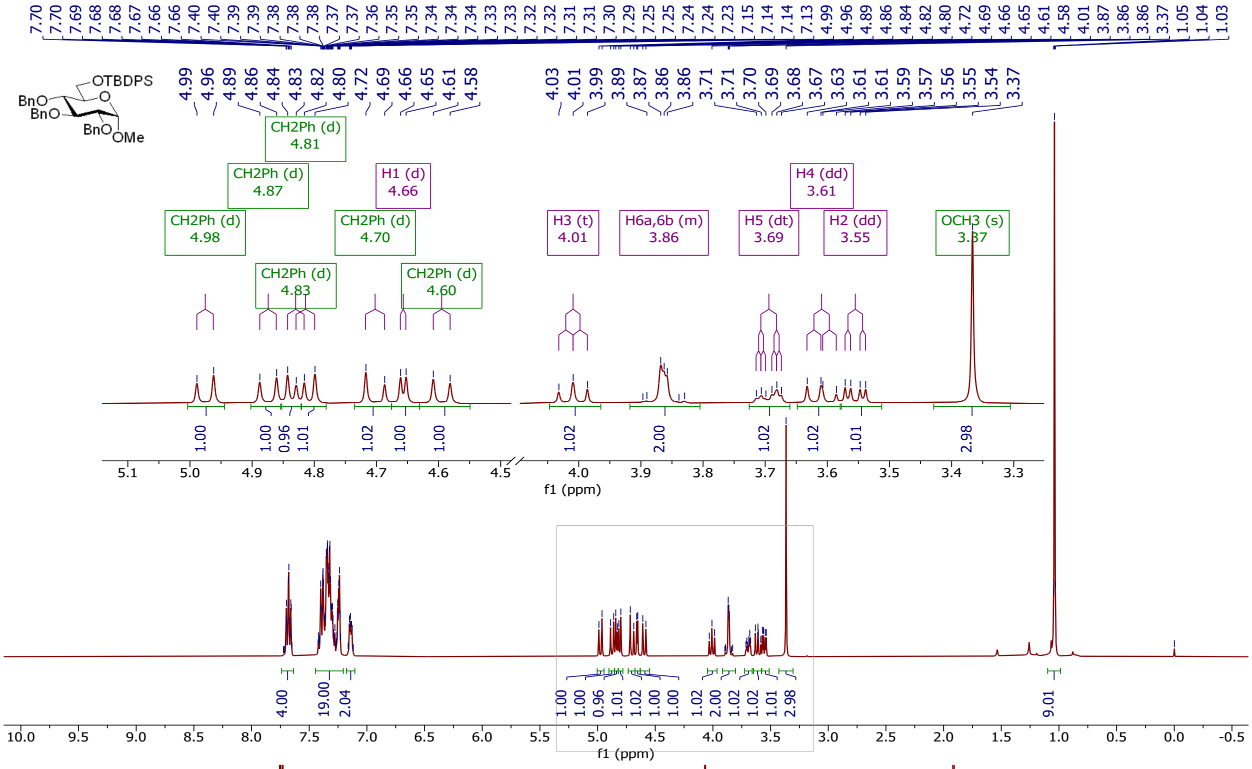

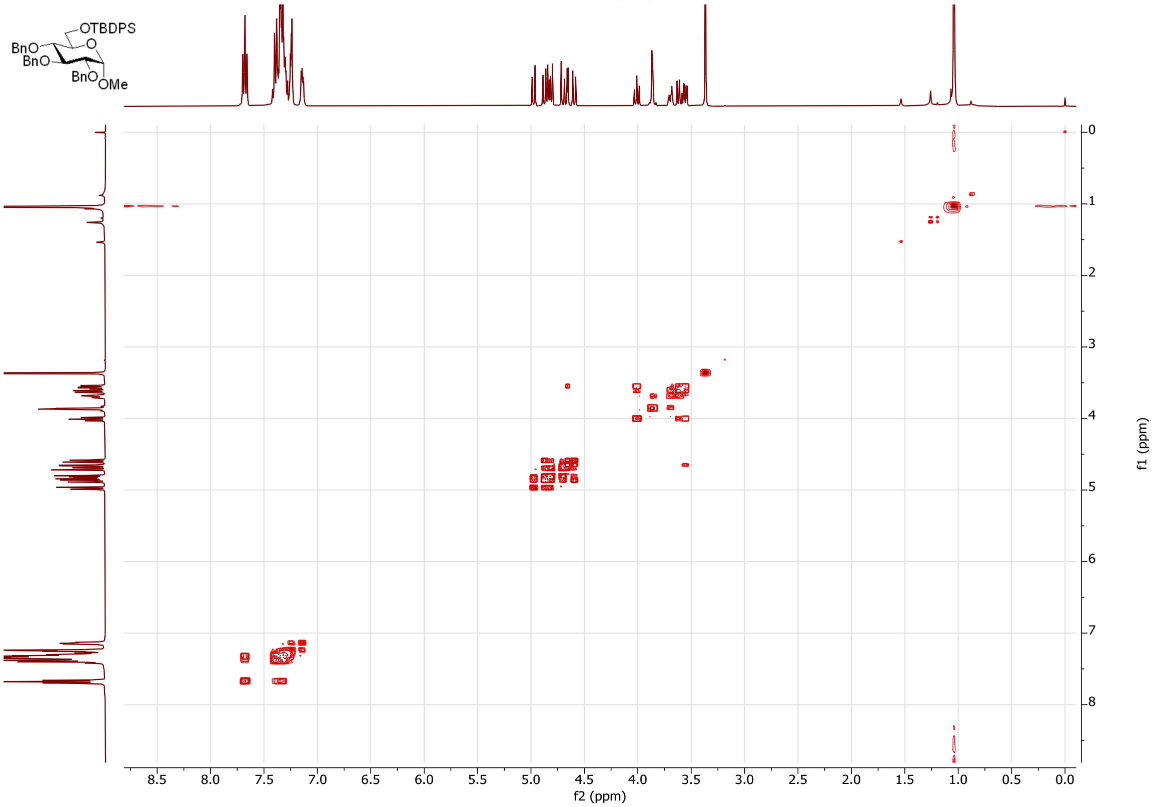

Methyl 2,3,4-tri-*O*-benzyl-6-*O*-*tert*-butyldiphenylsilyl-α-D-glucopyranoside (3).

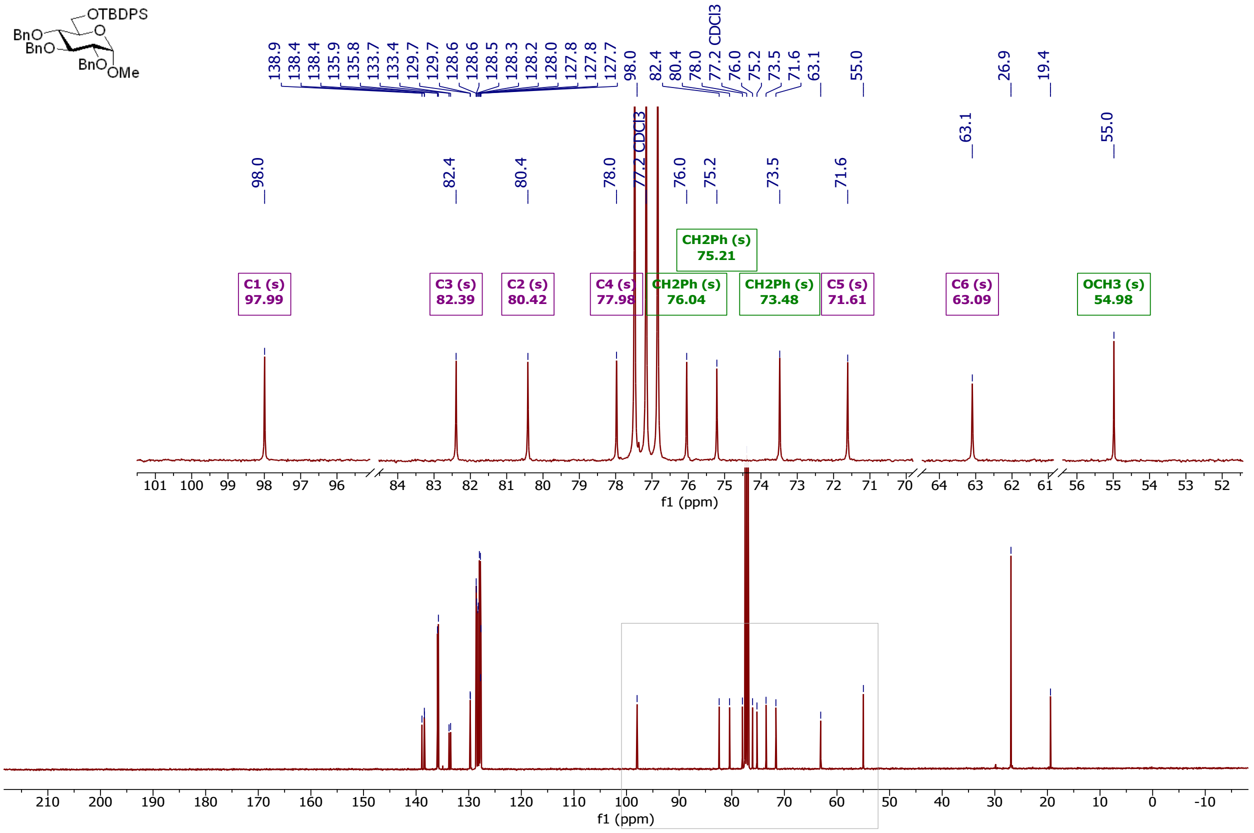

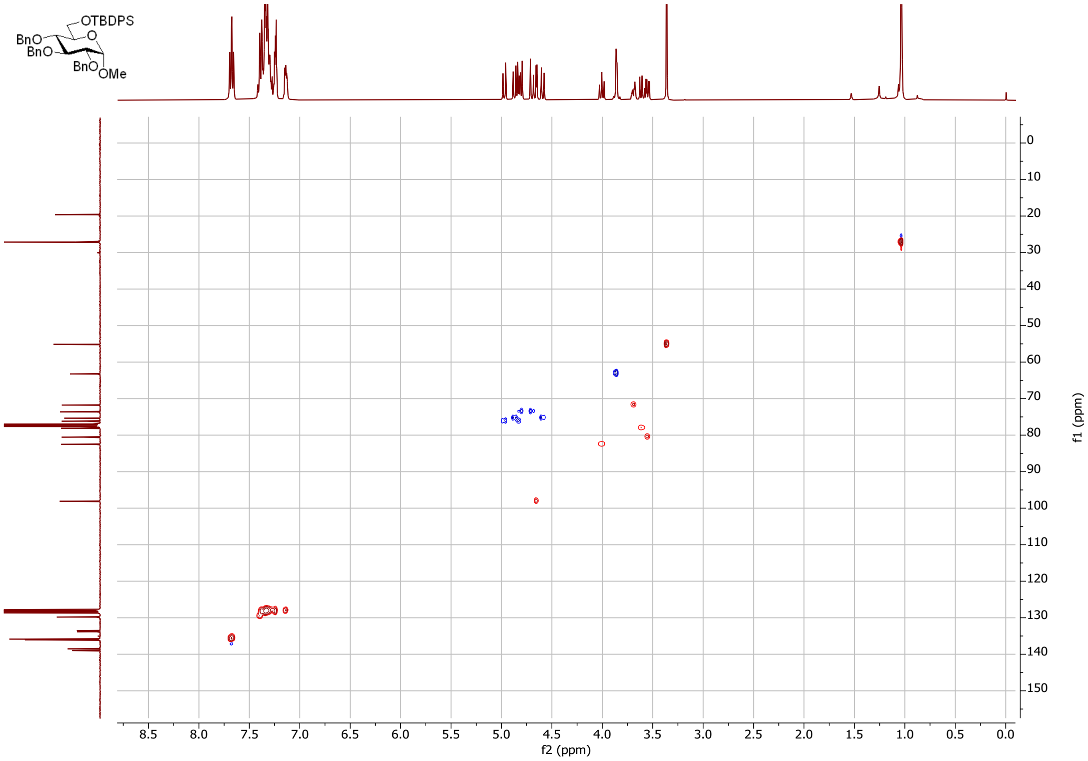

Methyl 2,3,4-tri-*O*-benzyl-6-*O*-*p*-nitrobenzoyl-α-D-glucopyranoside (5).

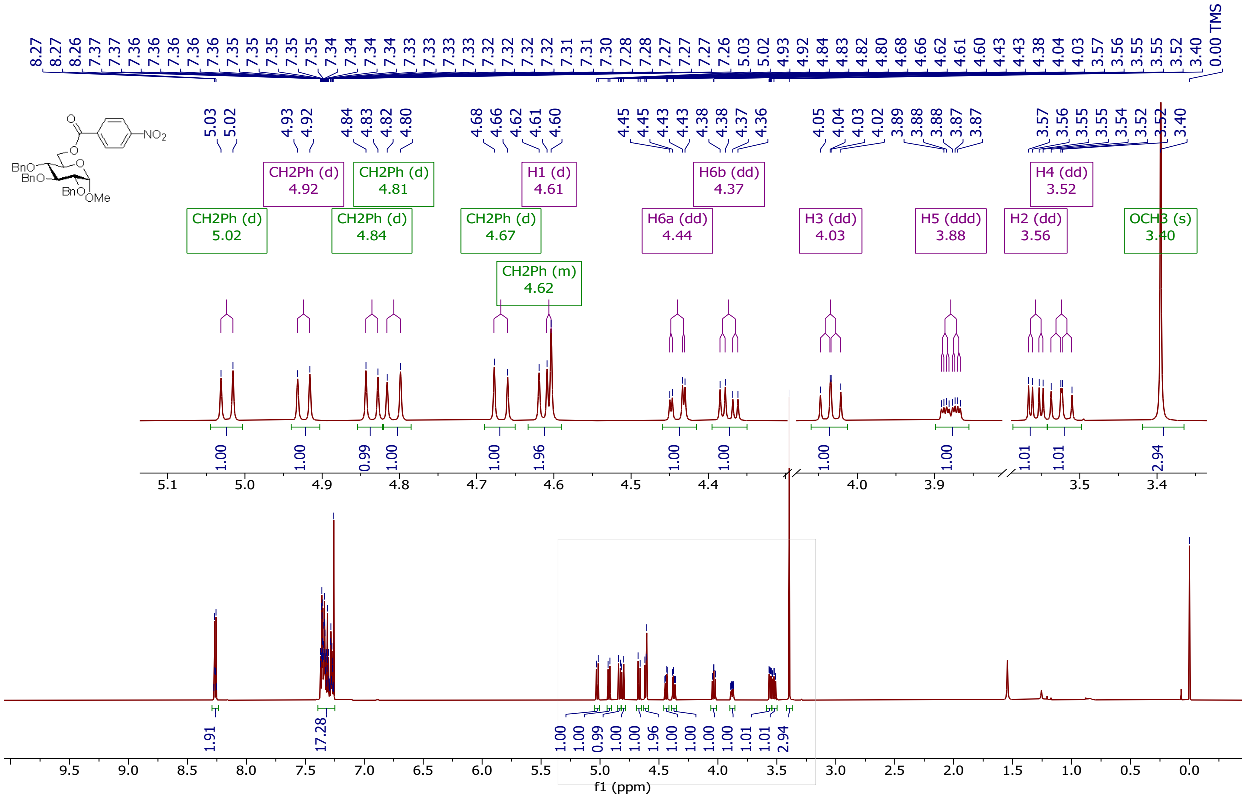

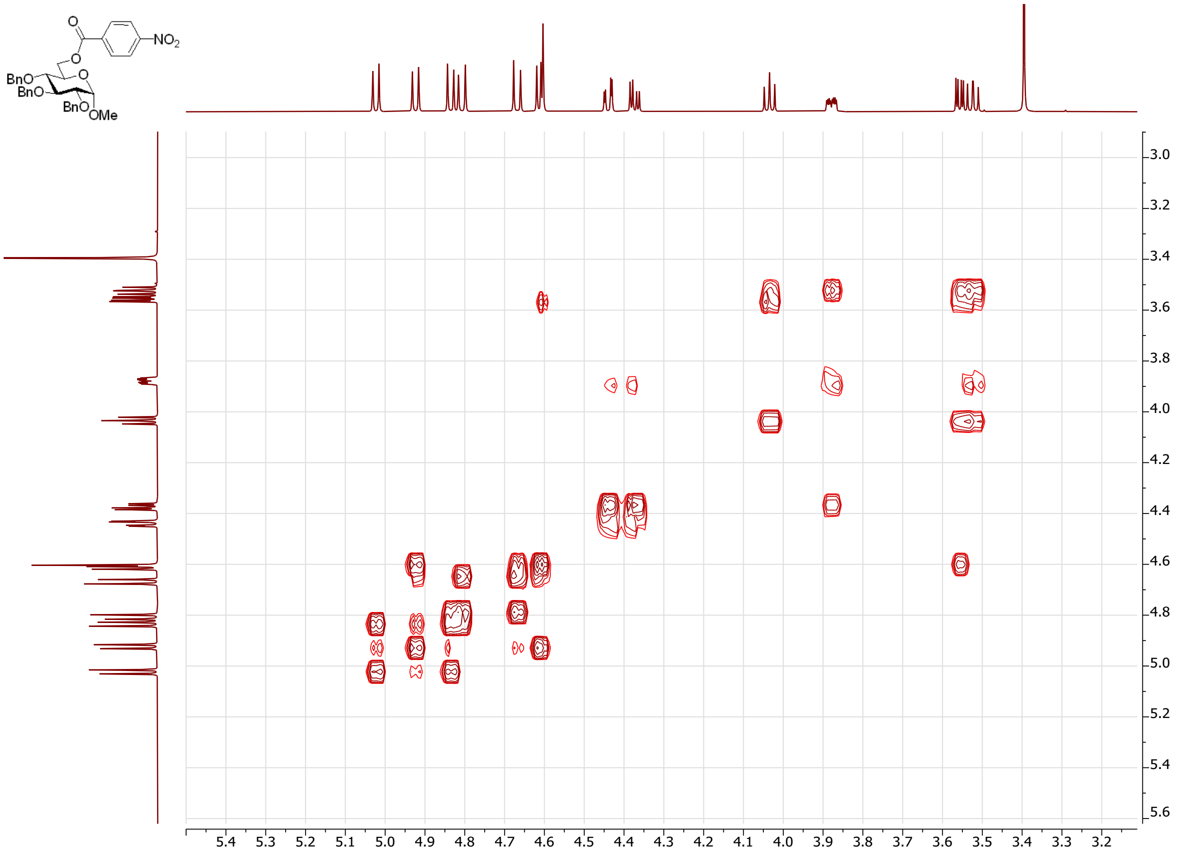

Methyl 2,3,4-tri-*O*-benzyl-6-*O*-*p*-nitrobenzoyl-α-D-glucopyranoside (5).

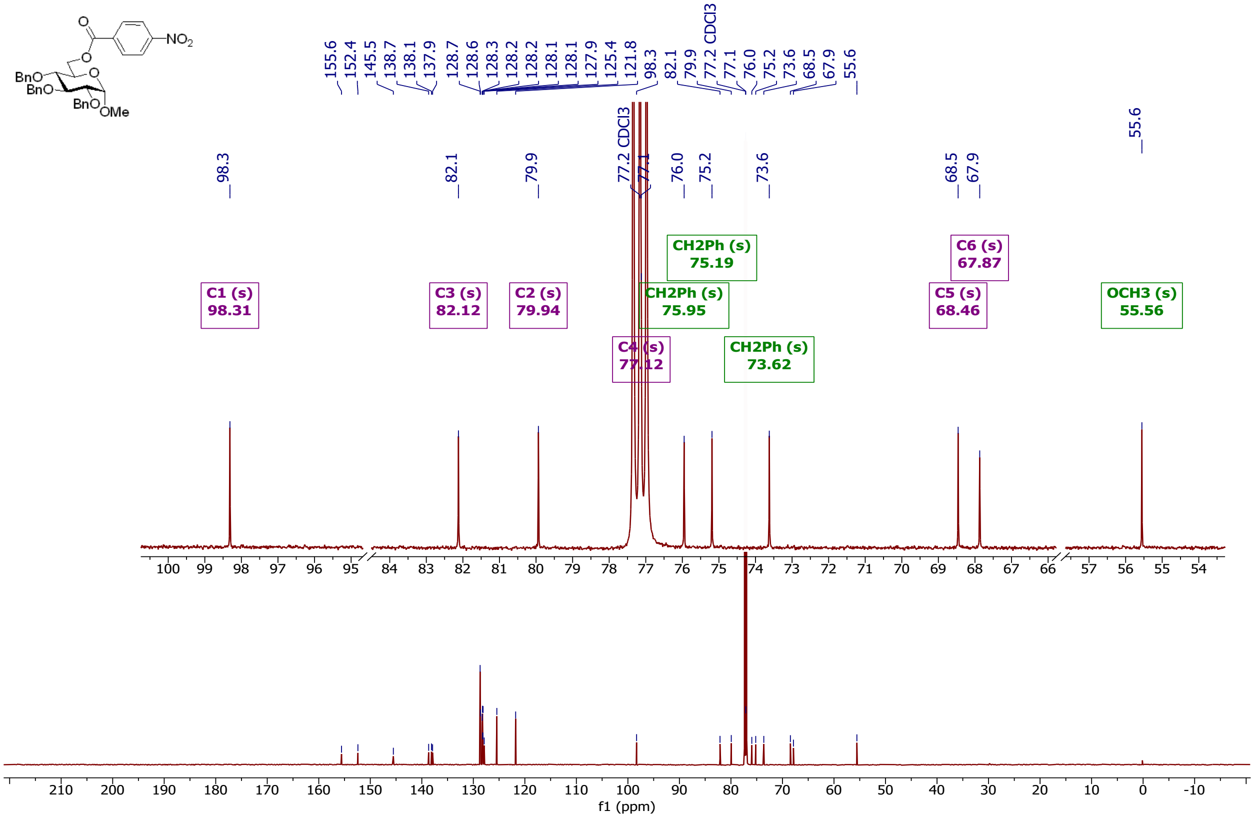

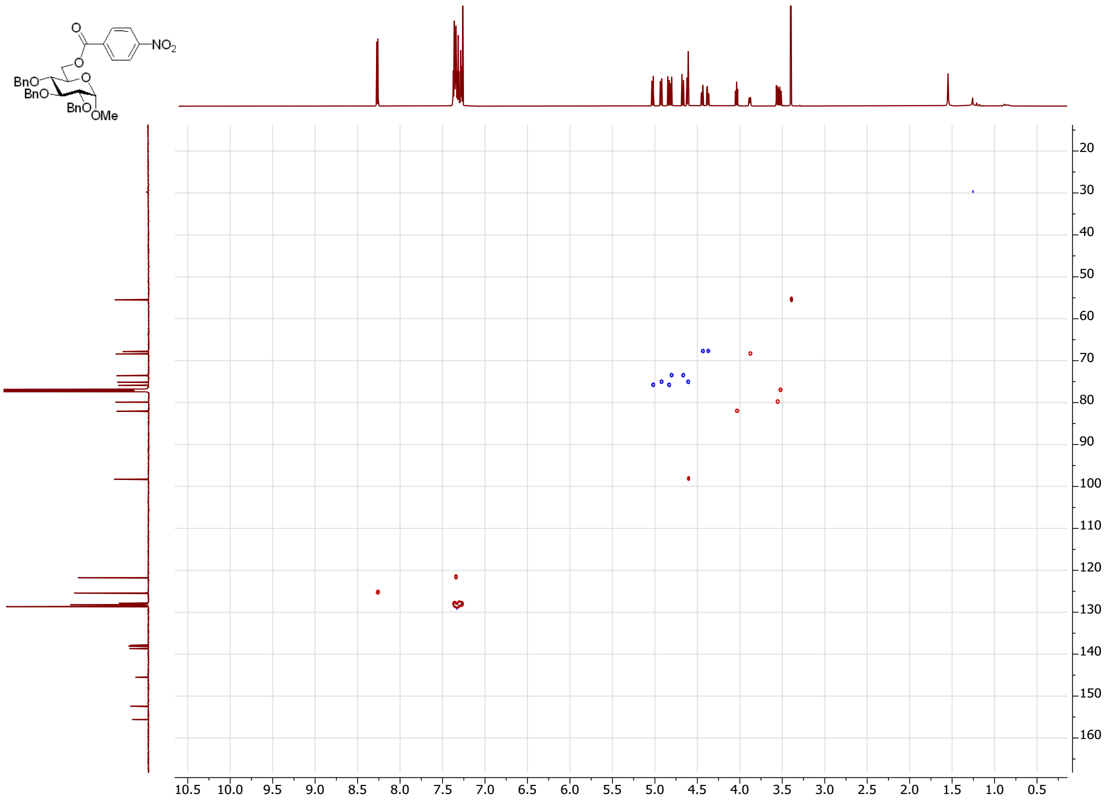

Methyl 2,3,4-tri-*O*-benzyl-6-*O*-*p*-methoxybenzoyl-α-D-glucopyranoside (6).

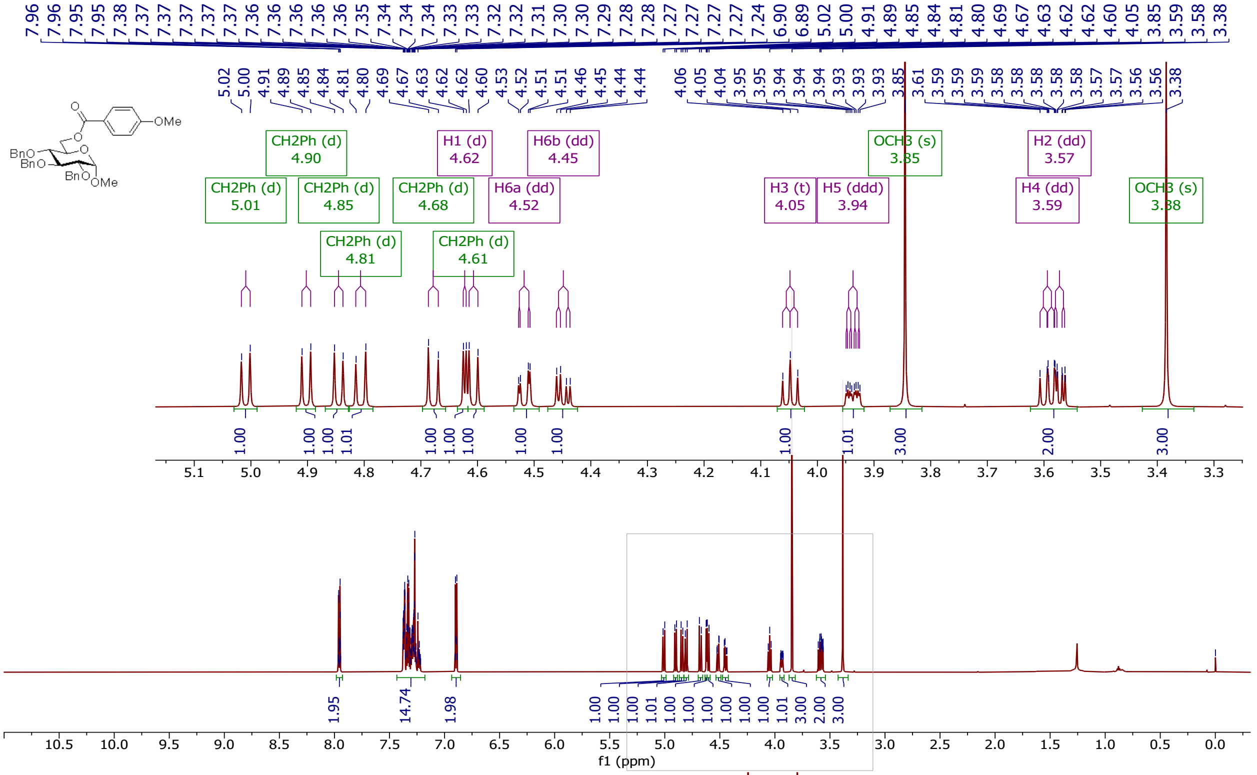

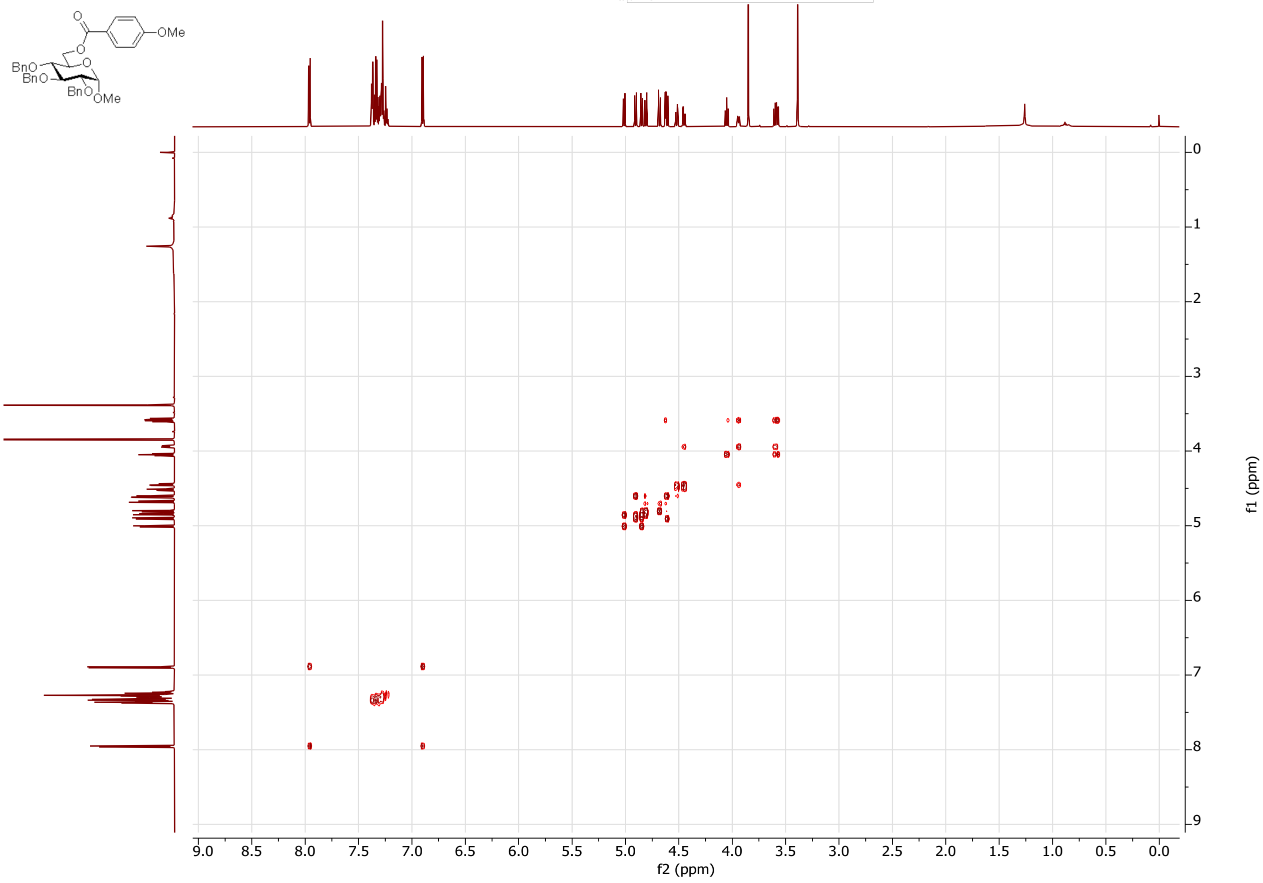

Methyl 2,3,4-tri-*O*-benzyl-6-*O*-*p*-methoxybenzoyl-α-D-glucopyranoside (6).

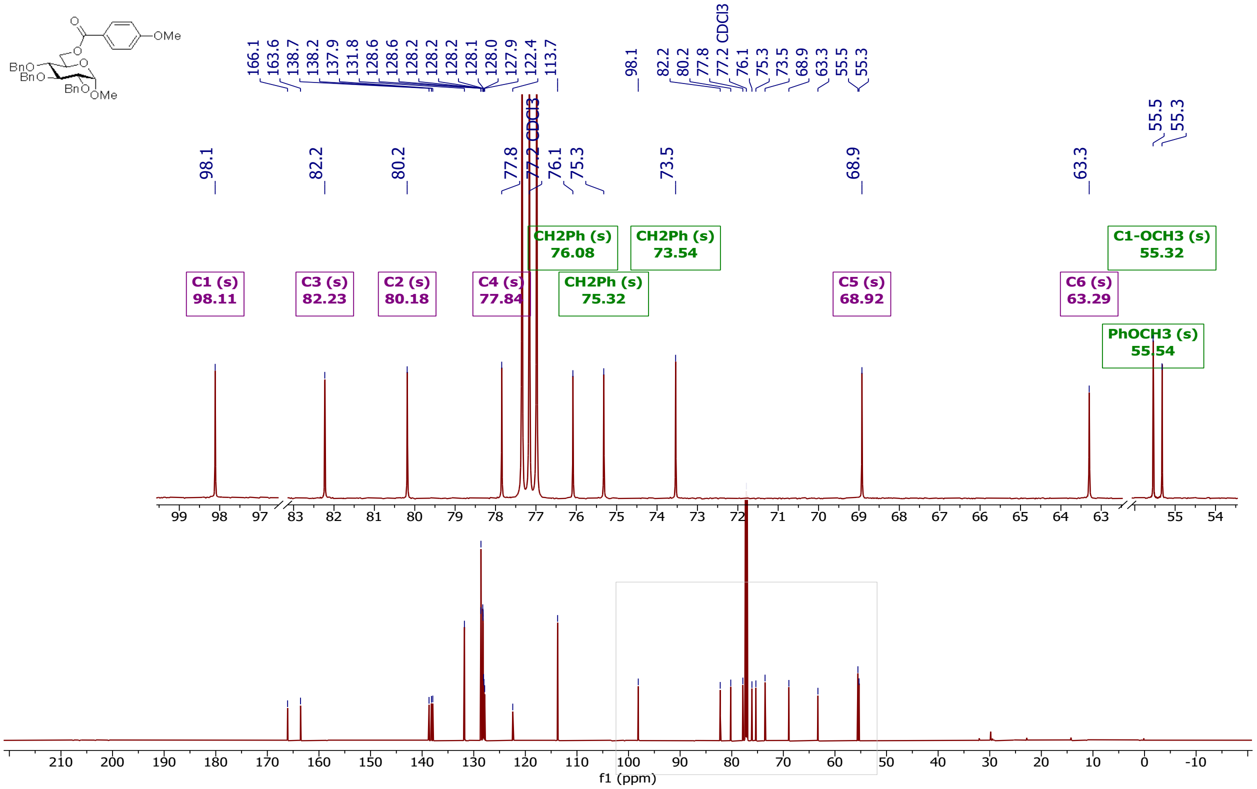

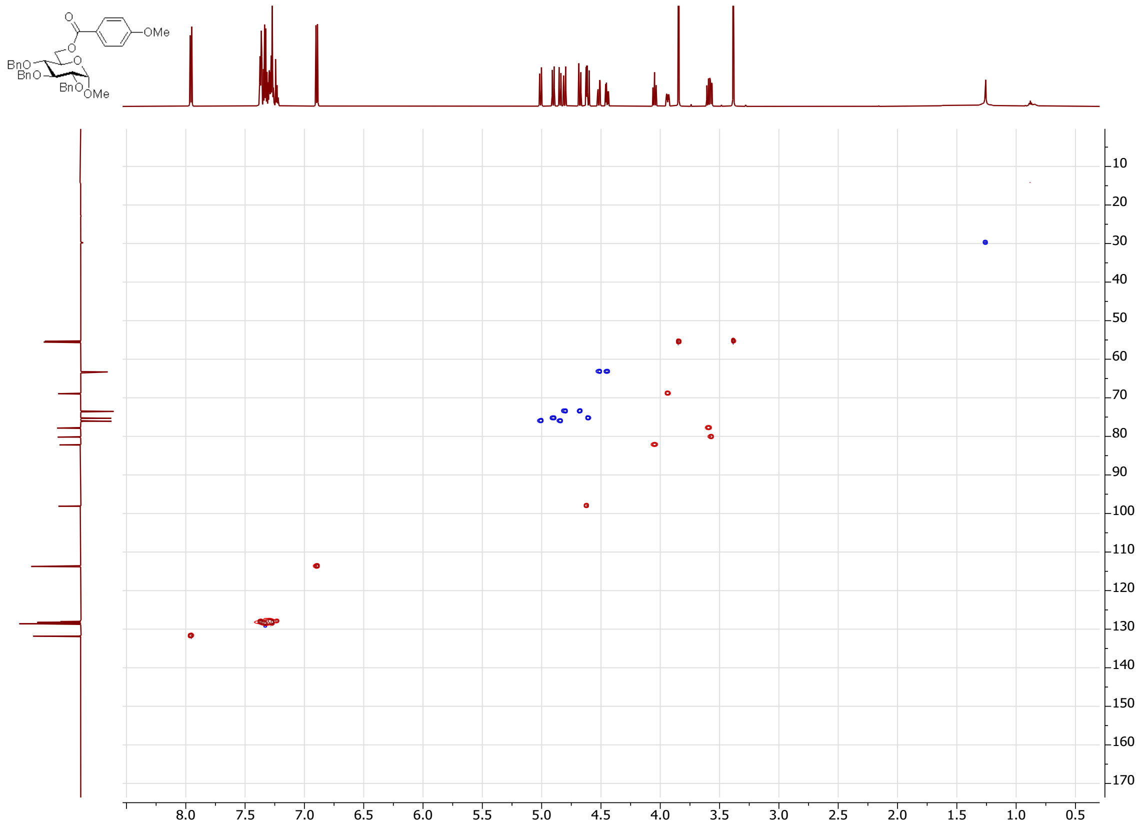

Supplement: SI [file NIHMS2163138-supplement-SI.pdf]
